# A Path Analysis Model Examining Factors Affecting the Caregiving Burden Experienced by the Family Caregivers of Drug Addicts in Egypt

**DOI:** 10.34172/jrhs.2022.89

**Published:** 2022-10-19

**Authors:** Marwa G Abdelrehim, Refaat R Sadek, Asmaa S Mehany, Eman S Mohamed

**Affiliations:** ^1^Public Health and Preventive Medicine Department, Faculty of Medicine, Minia University, Minia, Egypt; ^2^Medical Administration Unit, Minia University, Minia, Egypt

**Keywords:** Burden, Drug addicts, Family caregivers, Egypt

## Abstract

**Background:** Although the caregiving burden experienced by the family caregivers of drug addicts is receiving increased attention, there is still a need to study the possible predictors of the care burden, especially with the increasing numbers of addicts in Egypt and the important role of family caregivers in the support and treatment of addicts.

**Study Design:** A cross-sectional study

** Methods:** This study was conducted at Minia Hospital for Mental Health and Addiction Treatment, Egypt. Data was collected during interviews with addicts and their family caregivers. The caregiver burden was assessed using the Family Burden Interview Schedule (FBIS). The path analysis was used to assess the interrelationships between the burden and characteristics of addicts and caregivers.

**Results:** Based on the results, 96.7% of addicts were males, and their mean age was 28.8 ± 8.1 years, while their caregivers aged 39.7 ± 10.4 years and included 58.7% males. The caregivers reported a severe burden of care which was predicted by the addict’s drug-related problems (B=0.25, *P*=0.0003), financial hardship (B=0.46, *P*<0.0001), and the caregiver’s occupation (B=-0.16, *P*=0.017). Financial hardship had an indirect association with the burden of care (B=0.06, *P*=0.041) mediated through drug-related problems score, which was predicted by the severity of dependence, admission for treatment, and the level of social support.

**Conclusions: **The burden of caring for addicts depends on patient-related problems, as well as caregivers’ situations and income. Strategies to provide social support, financial aid, and problem-solving skills should be provided to the addicts and their caregivers as a part of treatment programs to help reduce the caregiving burden.

## Background

 Drug addiction is recognized as a devastating health and social problem in Egypt^1-6^ with a positive relationship with HIV and Hepatitis C prevalence.^7^ A large community-based survey among the Egyptian population above 15 years old found that 6.4% of cases met the criteria of drug addiction.^6^ Moreover, the prevalence rates of drug addiction in Egypt were 1.5% among secondary school students,^3^ 4.9%-8.1% among university students,^4,8,9^ 18.3% among industrial workers,^10^ and 21.5% among car drivers.^11^

 Family caregivers have a major role to play in supporting drug addicts, especially in developing countries where joint family and stronger familial bonds are more common.^12^ They help the drug addict to engage in the treatment, improve their health, and prevent relapses.^13^ Nonetheless, the provision of care for a drug addict has been associated with negative physical and emotional health outcomes for caregivers, diminishing their quality of life and satisfaction.^13,14^ Caregivers are at risk of various complications, such as stress, anxiety, depression, communication problems, financial problems, and social deprivation.^14^ Caregiver burden was defined as the level of multifaceted strain perceived by the caregiver from caring for a family member and/or loved one over time, including both subjective and objective outcomes.^15^

 Various factors, such as insufficient income, several responsibility conflicts, and decreased social activities, can affect the burden of caring for addicted individuals.^13,15^ Nevertheless, prior studies pointed out that the burden can be minimized after the implementation of a psychiatric nursing intervention program.^16^ Drug addiction-related family burden is an explicit problem in developing countries where joint family and stronger familial bonds are more common patterns compared to Western countries.^12^ Therefore, understanding the nature and predictors of caregiver burden has become necessary and serves as the basis for professional treatment and prevention interventions aiming at decreasing the caregivers’ burden and improving their physical and mental health, which in turn will positively affect the treatment and wellbeing of the addict.^13^ Considering the dramatically increasing number of drug addicts among young adults in Egypt in recent years and the lack of comprehensive research on the caregiver burden resulting from substance addiction, the current study aimed to verify a structural model of caregiver burden and assess its main predictors among the family caregivers of addicted patients.

## Methods

###  Study design and participants

 This cross-sectional study was carried out at Minia Hospital for Mental Health and Addiction Treatment (outpatient visits and hot-line clinic) for one year, from January 2020 to January 2021. All mentally sound outpatient addicts and their family caregivers, who were able to participate and provided written consent, were included in the study. Addict patients were excluded if they had a diagnosis of major chronic psychiatric illnesses (such as organic psychosis and schizophrenia) or serious medical conditions. Each addict was asked to identify his/her primary caregiver who was recruited if he/she meets the definition of a family caregiver and fulfills at least three of the criteria determined by Perlick et al: (1) a parent, partner, or another relative, (2) maintains frequent contact with the patient, (3) provides significant financial support to the patient, (4) accompanies the patient during consultation/treatment, aware of the severity of the illness, and supervises eating behavior at home, (5) is the person the therapy team contacts in the event of an emergency.^17^ A structured face-to-face interview, which lasted 30 minutes to 1 hour, was conducted separately for patients and their caregivers. The response rate to the study was approximately 90%. A total of 150 pairs of addicts and their family caregivers were recruited, which was a suitable sample size for the path analysis. Considering the number of independent variables in the study (N-1) and a medium effect size ( < 0.05), the required sample size was 125.^18^

###  Data collection and measures 

 The investigator collected sociodemographic information, including age, gender, level of education, and occupation. Caregivers were asked about their relationship with the patient, duration of care, and financial hardship. They completed the Family Burden Interview Schedule (FBIS) to measure the burden of care for addicts. Nonetheless, addict patients were asked to complete the Severity of Dependence Scale (SDS), Drug Abuse Screening Test-20 (DAST-20), Perceived Devaluation and Discrimination Scale (PDD), and Social Support Questionnaire short-form (*SSQ6*).

###  Statistical analysis

 All analyses were performed in SAS software (version V9.4). The first step included descriptive statistics of sociodemographic variables which were presented as mean and standard deviation for quantitative data, as well as frequencies and percentages for categorical variables. The reliability of the study scales was assessed with Cronbach’s alpha coefficient ( ≥ 0.70 is sufficient). The second step involved the bivariate correlation analyses between the caregiver burden (FBIS score) and all possible predictors in the study. The design of the initial model was based on the findings of previous studies and the significant bivariate associations of the present study. Categorical variables (education and occupation) were used as binary variables in the path analysis.

 The third step aimed at verifying the model of the caregiver burden based on theoretical and empirical assumptions. The goodness-of-fit of the model was assessed through the accepted norms of different statistical and psychometric indices: Chi-square test *P* > 0.05, goodness-of-fit index (GFI) ≥ 0.85, adjusted goodness-of-fit index (AGFI) ≥ 0.80, and root mean square error of approximation (RMSEA) ≤ 0.08, standardized root mean square residual (SRMR) ≤ 0.10, and Bentler’s comparative fit index (CFI) ≥ 0.90. Moreover, the fit of path coefficients was tested, and the absolute value of t > 1.96 was accepted as significant. Changes to the initial model were based on the standardized residual matrix, the significance of the causal path, and the modification indices which included the Wald test and Lagrange multiplier test.^18,19^

## Results

 The majority of addict patients (96.7%) were males and dependent on two or more illicit drugs, including cannabis, alcohol, tramadol, heroin, strox, and other opioids. The mean age of addicts was 28.8 ± 8.1 years, 13.4% had a university education, and more than half of them (51.3%) were single. The mean duration of addiction was 4.5 ± 2.8 years, and the majority of patients (84%) underwent hospital admission for treatment. On the other side, 58.7% of the caregivers were males, and their mean age was 39.7 ± 10.4 years. Moreover, 30.7% of the caregivers were parents, 80% were married, and 68% reported financial hardship during the period of caregiving, as displayed in Table 1.

**Table 1:** Sociodemographic characteristics of addict patients and their family caregivers(n = 150)

**Categorical variables**	**Addict patients**		**Family caregivers**	
	**Number**	**Percent**	**Number**	**Percent**
Gender				
Male	145	96.7	88	58.7
Female	5	3.3	62	41.3
Education				
Illiterate	21	14	16	10.7
Primary	44	29.3	29	19.3
Secondary	65	43.3	68	45.3
University and above	20	13.4	37	24.7
Occupation				
Non-worker	61	40.7%	51	34%
Manual worker	50	33.3	66	44
Clerk	39	26	33	22
Marital status				
Single	77	51.3	14	9.4
Married	60	40	120	80
Divorced or widow	13	8.7	16	10.6
Residence				
Urban	81	54	No data	No data
Rural	69	46	No data	No data
Hospital admission for treatment				
Yes	126	84	No data	No data
No	24	16	No data	No data
Relation to patients				
Partner	No data	No data	36	24
Parent	No data	No data	46	30.7
Sibling	No data	No data	37	24.7
Other	No data	No data	31	20.6
Living with patient				
Yes	No data	No data	59	39.3
No	No data	No data	91	60.7
Financial hardship				
Yes	No data	No data	102	68
No	No data	No data	48	32
**Continuous variables**	**Mean**	**SD**	**Mean**	**SD**
Age (y)	28.8	8.1	39.7	10.4
Duration of addiction (y)	4.5	2.8	No data	No data
Duration of care (y)	No data	No data	4.0	2.3
Time of care (h/wk)	No data	No data	67.8	60.1

 The scales used in the study had good internal consistency with Cronbach’s alpha coefficient ≥ 0.70 (Table 2). The addict patients reported high psychological dependence on drugs (SDS mean score was 11.4 ± 1.9), as depicted in Table 2. They reported substantial/severe drug-related problems and perceived discrimination from the public (DAST-20 and PDD mean scores were 15.1 ± 2.6 and 2.8 ± 0.4, respectively). On the other hand, the caregivers reported a severe burden of care (FBIS mean score was 37.1 ± 7.4) whichwas positively correlated with drug-related problems (DAST-20score).

**Table 2:** Means, standard deviations (SD), ranges, Cronbach’s alpha, and inter-correlations for scores of the study scales

**Measure**	**Mean (SD)**	**Range**	**Cronbach's alpha**	**Pearson's Correlation **			
				**2**	**3**	**4**	**5**
1. Severity of dependence scale (SDS)	11.4 (1.9)	6-15	0.70	0.19*	0.11	-0.08	0.07
2. Drug Abuse Screening Test (DAST–20)	15.1 (2.6)	8-20	0.71		0.10	-0.18^a^	0.32^b^
3. Perceived Devaluation Discrimination scale (PDD)	2.8 (0.4)	1.9-3.8	0.88			-0.17^a^	0.11
4. Social Support Questionnaire short form (SSQ6)	5.8 (2.3)	2-10	0.79				-0.08
5. Family Burden Interview Scale (FBIS)	37.1 (7.4)	7-48	0.89				

^a^ Correlation is significant at the *P* < 0.05 level (2-tailed). ^b^ Correlation is significant at the *P* < 0.001 level (2-tailed).

 Regarding the financial burden, a severe burden was reported due to the loss of addicts’ income (43.3%) and the loss of other family members’ income due to patients’ illness (31%). Moreover, the disruption of routine activities of family members, family leisure, and family interaction due to patients‘ illness led to a severe burden in 60.7%, 44%, and 57.7% of participants, respectively. Physical and psychological illnesses of family members brought on by patients’ behavior were found among 72.7% and 83.3% of cases, respectively

**Figure 1:**
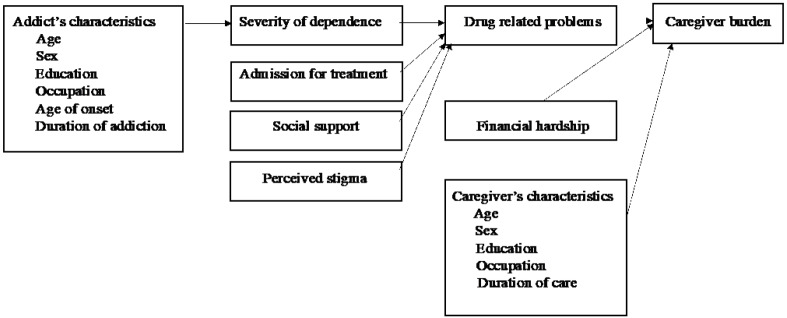
Initial model of the burden of care among caregivers of addicts

 The initial model for caregiver burden (Figure 1) was specified based on previous findings and the significant bivariate correlation analyses among study variables. In the initial model, the main outcome was the caregiver burden (FBIS score), while drug-related problems (DAST-20), caregiver’s characteristics (age, gender, education, occupation, and duration of care), and financial hardship were treated as primary predictors for burden. Moreover, a secondary outcome, drug-related problems score (DAST-20), was considered and was dependent on the severity of dependence (SDS score), admission for treatment, perceived discrimination (PDDscore), and perceived social support (SSQ6score). Finally, the addict’s characteristics (age, gender, education, occupation, age of onset, and duration of addiction) predicted the severity of drug dependence (SDS score)as illustrated in the initial model (Figure 1).

**Figure 2:**
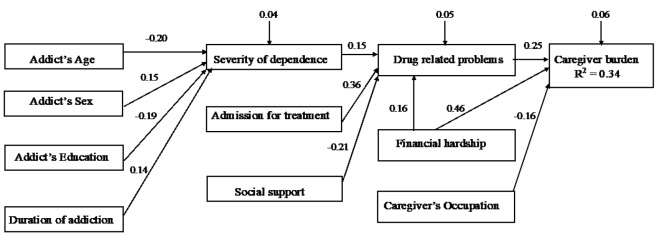
Reduced model of burden among caregivers of addict patients. Standardized path coefficients and error values were presented. Covariates are omitted from the figure for pictorial purposes

 On the basis of path analysis, the final model was presented in Figure 2 and Table 3. The fit of the overall model was acceptable based on different statistical indices: Chi-square test *P* = 0.897, GFI = 0.989, AGFI = 0.954, RMSEA = 0.00, SRMR = 0.026, and CFI = 1.00. Moreover, all path coefficients were significant. The final model demonstrated significant direct associations of the caregiver burden with the patient’s drug-related problems (B = 0.25, *P* = 0.0003), financial hardship (B = 0.46, *P* < 0.0001), and caregivers’ occupation (B = -0.16, *P* = 0.017) as shown in Figure 2 and Table 3. The findings suggest that an increase of 1 SD in the drug-related problems score was associated with an increase of 0.25 SD in the caregiver burden score. The presence of financial hardship in the family was associated with an increase of 0.46 SD in the caregiver burden score, and caregivers’ employment was associated with a decrease of 0.16 SD in the burden of care score.

**Table 3:** Regression estimates (with beta values) in the final path analysis model

**Path**		**Unstandardized regression coefficient**	**Standard error**	**t-value**	**Standardized regression coefficient**
**From**	**To**				
Drug-related problems	Burden of care	0.69	0.19	3.49	0.25
Financial hardship	Burden of care	7.21	1.08	6.64	0.46
Caregiver's occupation	Burden of care	-2.49	1.05	2.36	-0.16
Severity of dependence	Drug-related problems	0.20	0.09	1.99	0.15
Admission for treatment	Drug-related problems	2.49	0.51	4.85	0.36
Social support	Drug-related problems	-1.16	0.42	-2.76	-0.21
Financial hardship	Drug-related problems	0.89	0.40	2.21	0.16
Addict's age	Severity of dependence	-0.06	0.02	-2.34	-0.20
Addict's sex	Severity of dependence	1.64	0.82	1.98	0.15
Addict's education	Severity of dependence	-0.71	0.30	-2.36	-0.19
Duration of addiction	Severity of dependence	0.10	0.06	1.98	0.14

 In addition to the significant direct association between financial hardship and caregiver burden, an indirect association was observed (B = 0.06, *P* = 0.041) mediated through drug-related problems. Furthermore, there were significant associations between drug-related problems and the severity of dependence, admission for treatment, and level of social support. The severity of drug dependence was predicted by the addict’s age, gender, education, and duration of addiction, as presented in Table 3. The combined set of variables explained 34% of the variance in caregiver burden. Although no direct impact was observed on the caregiver burden, the final model pointed to the significant indirect association of the burden of care with the severity of dependence (B = 0.04, *P* = 0.042), the perceived social support (B = –0.06, *P* = 0.028), and admission for treatment (B = 0.10, *P* = 0.004).

## Discussion

 The provision of care to an addicted relative can lead to physical, psychological, and social alterations in the caregiver’s life. The current study found that caregivers of addicted patients at Minia Hospital for Mental Health and Addiction Treatment were likely to experience a severe burden of care predicted by drug-related problems, financial hardship, and the caregiver’s occupation.

 The demographic attributes (age, gender, education, and occupation) of the studied addicts were generally similar to what was reported in earlier studies among addicts in Egypt^4,5,8,20-22^ and other countries.^23-26^ The patients in the current study were mostly males (96.7%), single (51.3%), and employed (59.3%); nonetheless, only 13.4% of cases had a university education. However, a recent study among addicts in the Heliopolis psychiatric hospital in Cairo reported that 86.7% of subjects were males, 43.3% had a university education, and 6.7% were single.^20^ Another study in Turkey found that 78.9% of addicts were males, 60.5% were single, and 43.4% were employed.^27^ These differences can be ascribed to the effect of the location of this study in Upper Egypt, where conservative traditions and possible stigma reduce the prevalence of addiction and the demand for hospitalized treatment. These discrepancies can also be attributed to the small sample size of the latter studies.

 On the other side, the demographic features of the studied caregivers differ from what was reported in previous similar studies.^23,27^ The current caregivers were younger (mean age was 39.7 ± 10.4 compared to 43.32 ± 13.35 in Turkey and 45.85 ± 12.92 in India), mainly males (58.7% compared to 30.3 % in Turkey and 32% in India), generally employed (66% compared to 38% in India), with secondary or higher education (70% compared to 52.6 % in Turkey and 40.6% ).^23,27^ Despite these differences and their better situation, the caregivers in the current study reported a severe burden of care reflecting the difficulty in the caregiving role they undertook.

 As expected, in accordance with most studies,^12,27,28^ the findings of this study revealed a severe burden of care among the family caregivers of addict patients. At the same time, one study showed a lower mean FBIS score in the objective burden (20.5 ± 6.4)^23^ compared to that in the current study (37.1 ± 7.4). Caregivers of drug addicts have a lot of negative feelings, such as sadness, anger, stress, and guilt.^14^ They experienced negative physical and psychosocial consequences of the caregiving role, such as musculoskeletal disorders, sleep disturbance, anxiety, stress, and social isolation. Caregivers also expressed financial constrain and feelings of shame while taking credits from others to fulfill their financial needs,^14,28^ signifying a severe caregiver burden, especially in developing countries, such as Egypt.

 Drug-related problems, assessed by DAST-20 and reported by the studied addicts, were among the key predictors of caregiver burden. Substance use disorders are usually associated with high levels of anxiety, depression, and violence which increase the severity of drug-related problems.^5,21^ Financial hardships were also significantly associated with caregiver burden both directly and indirectly mediated by drug-related problems. This finding was in line with most literature that emphasized that low family income^12,23,29^ and poor economic status^25^ increase the burden of caring for addicted patients. Caregivers usually need funds for the treatment and care of the addict, which is a costly issue worldwide. Moreover, they may have a feeling of shame that prevent taking money from others and can lead to financial hardship.^12,14^

 Inconsistent with some previous studies, which reported no significant association between the caregiver’s occupation and the burden of care,^12,23^ the findings of the present research pointed out that the caregiver’s occupation can predict the burden of care and unemployment increases the caregiving burden. A possible explanation is that more than half of the currently studied caregivers were males, and unemployment leads to spending most of the time at home burdened with the addicts’ problems and caregiving tasks, especially if the patients were not working, apart from the associated financial constraints that further increase the burden.^14^

 In agreement with previous findings, the majority of addicts in the present study were dependent on more than one class of substances and were highly dependent on drugs.^4,5,8,26^ The severity of dependence was significantly associated with drug-related problems and indirectly related to the caregiver burden. These findings support other studies in which a higher level of drug addiction was significantly associated with higher depressive symptoms, lower family relations,^30^ more drug abuse-related problems, and consequently, the higher burden of care among their relatives.^23,31,32^ While this study pointed out that the addict’s age, gender, education, and duration of addiction predicted the severity of drug addiction. A high level of dependence was associated with younger age, male patients, lower education, and longer duration of addition.

 Although this study did not point to the direct impact of social support of the addict on the caregiver burden, its effect was manifested indirectly through drug-related problems. The study by Dağlı et alreported that caregivers perceived the social and recreational activities of their related addicts to be inadequate.^27^ Moreover, Ahmad et al revealed a significant positive relationship between social support and the quality of life among the caregivers of drug-addicted people.^33^ Social relationships were among the most affected aspects of life in the whole family of addicted patients due to reduced opportunities for leisure activities, changes in family routines, and difficulties encountered in working and meeting their colleagues.^33^ Moreover, some addicts and caregivers chose social isolation as a tool to safeguard their reputation in society by avoiding interaction and outreach, which can increase depression and the burden of caregiving.^14^

 Consistent with previous results,^23^ the previous admission for treatment was among the predictors of drug-related problems and indirectly related to the caregiver burden. A recent study in Egypt indicated that young addicts aged 25-35 were most prone to multiple craving factors and inability to cope with interpersonal problems, inability to find a suitable job, or lack of family and social support,^20^ which can prolong and hinder effective treatment and increase the burden of family caregiving. Increased burden of care for addicts may affect treatment compliance and causes overall poor quality of life for both patient and family members. Therefore, assessing the burden of care for drug-addicted patients, a group counseling approach based on the quality of life, and developing caregivers’ coping skills are highly recommended to prevent and treat problems, reduce stress, and improve mental health and life satisfaction among family caregivers of drug addicts.^24,32,34^

 Among the notable strengths of this study, we can refer to the use of validated instruments and the long time allocated to data collection (one year) that enabled interviewing a large number of patients and their family caregivers, easy obtaining of information, and reliable assessment. Furthermore, the study evaluated both caregiver and patient factors and used the path analysis model to clarify the direct and indirect relationships between the factors affecting the burden of caregiving for addicts. However, the study has some limitations that should be considered. This is a cross-sectional study that was based on self-reported data provided by patients and their caregivers; therefore, there was a potential for reporting bias and an inability to make statements about causation. Another limitation is that we did not examine additional factors, such as chronic diseases, life events, satisfaction level, coping skills, type of drug, and program of treatment, that may affect the caregiver’s burden. Moreover, the findings presented in this study cannot be generalized since it included patients who seek treatment for addiction in a single hospital limited to a specific region, not the whole community of drug addicts. Therefore, future multicultural research in different geographical locations should be conducted with a prospective design to study the burden of care imposed on the caregivers of addicts and all the possible factors influencing it.

**Highlights**

- Addicts reported high drug dependence and substantial/severe drug-related problems.
- Caregiver burden was predicted by drug-related problems and caregivers’ occupation.
- Financial hardship exerted direct and indirect negative effects on the caregiving burden.
- The severity of drug dependence and admission for treatment affected the burden of care indirectly.
- Perceived social support had a significant indirect association with the burden of care.

## Conclusion

 In conclusion, the caregivers of addicted patients suffer from a severe burden that can be predicted by both addict-related problems, as well as the caregiver’s occupation and finance. There were also indirect effects of the severity of dependence, the perceived social support, and hospital admission for treatment on the burden of care. Therefore, the provision of social and financial support to the addicts and their caregivers, as well as facilitating self-help and problem-solving programs, may reduce the burden of providing care to addicts.

## Conflict of interest

 None declared.

## Ethical approval

 This study was approved by the Ethical Committee of Research of the Faculty of Medicine, Minia University and by the General Secretary of Mental Health and Addiction treatment of the Ministry of Health and Population.

## Funding

 No funding was received.
